# Jumping Kinematics and Performance in Fighting Crickets *Velarifictorus micado*

**DOI:** 10.3390/biomimetics11010049

**Published:** 2026-01-07

**Authors:** Yun Xing, Yan Zhang, Yu Yan, Jialing Yang

**Affiliations:** 1 Beijing Advanced Innovation Center for Materials Genome Engineering, Corrosion and Protection Center, Institute for Advanced Materials and Technology, University of Science and Technology Beijing, Beijing 100083, China; xingyun@ustb.edu.cn (Y.X.); m202421685@xs.ustb.edu.cn (Y.Z.); yanyu@ustb.edu.cn (Y.Y.); 2 Institute of Solid Mechanics, School of Aeronautic Science and Engineering, Beihang University, Beijing 100191, China

**Keywords:** insect, locomotion, jumping performance, power output, bio-inspired design

## Abstract

Jumping is a fundamental locomotion in insects, offering high performance and efficient movement. However, the relationships between the jumping force and performance remain inadequately understood. Here, we combine experimental measurements with a theoretical model to investigate the jumping kinematics and performance of crickets *Velarifictorus micado*. We examine how jumping force, gravity, aerodynamic drag, and take-off angle influence the jumping velocity, displacement, and power output of the crickets. We discuss the mechanistic advantages of various jumping force designs and demonstrate that the front slow-loaded force adopted by crickets enables greater power output while minimizing take-off displacement and acceleration time. The results show that aerodynamic drag exerts negligible influence, whereas gravity mainly affects the vertical propulsive component during the take-off phase. The gravitational effect leads to a decrease in resultant velocity and displacement with increasing take-off angle. This study advances our understanding of the mechanical principles governing jumps of insects and provides valuable insights for the design of high-performance jumping robots and catapult systems.

## 1. Introduction

Locomotion is a key evolutionary adaptation enabling animals to interact with their environments [1]. Among various locomotor modes, jumping is a widespread and efficient strategy in insects, facilitating essential biological functions such as predation, evasion, obstacle negotiation, and intraspecific competition [2,3,4,5,6]. Understanding the jumping mechanics of insects may provide inspiration for the design of high-performance artificial mechanical systems [7,8].

Crickets (Orthoptera, Gryllidae) are notable for their exceptional agility and jumping capability [9,10]. In China, there has been a traditional culture of crickets fighting since the Song dynasty (AD 960–1279) [11]. Fighting crickets *Velarifictorus micado* exhibit remarkable jumping performance and can maintain postural stability during the aerial phase, without any aerodynamic assistance from the wings. The researchers explored the functional importance of the hind legs in crickets, particularly in the context of their locomotor and survival behaviors [12,13,14,15,16]. Through a combination of experimental, theoretical, and finite element analyses, Xing and Yang [17] elucidated that *V. micado* dynamically manipulate aerodynamic force through strategic adjustments of their hind legs during the aerial phase, enhancing jumping stability and cushioning landings. Nevertheless, the jumping kinematics and performance of the crickets during the take-off phase are not fully understood.

Increasing theoretical effort has been devoted to unraveling the jumping mechanisms of insects [18,19,20]. Bennet-Clark [21] quantified the energy and power output required during the take-off phase of locusts *Schistocerca gregaria* (Orthoptera, Acrididae). Alexander [22] established mathematical models to study the effects of muscle properties, leg design and jumping strategies on the jump height. By considering the effect of flexibility on jumping responses, Chen et al. [23,24] proposed a flexible–rigid mechanical model to investigate the jumping process and kinematic characteristics of locusts. Burrows and co-authors studied the jumping mechanisms in other Orthoptera, including mole crickets [25] and bush crickets [26]. Patek et al. [27] used theoretical methods to estimate the force, torque, and trajectory generated by the trigger hairs on the mandibles of trap-jaw ants *Odontomachus bauri* (Hymenoptera, Formicidae). Nadein and co-authors investigated the jumping mechanisms in three beetles, including the marsh beetle (Coleoptera: Scirtidae) [28], weevil (Insecta: Coleoptera: Curculionidae) [29], and flea beetle (Coleoptera: Chrysomelidae: Alticini) [30]. These studies elucidated the biomechanical principles and elastic energy mechanisms underlying beetle jumping, offering valuable insights for bioinspired design. Ilton et al. [31] elucidated the underlying principles of jumping power amplification, highlighting how small organisms achieve extraordinary accelerations. Expanding upon the concept of jumping power amplification, Longo et al. [32] developed the latch-mediated spring actuation (LaMSA) framework, a comprehensive analytical method to explain various rapid biological motions across insects, plants, and bio-inspired robotics. However, existing theoretical approaches remain insufficient to clarify the mechanistic relationships between the jumping force and performance in insects.

In this study, we combine experimental measurements and theoretical modeling to investigate the jumping kinematics and performance of crickets *Velarifictorus micado*. We first examine the body morphology and microstructures by using microscopic analysis. The take-off process and jumping performance of *V. micado* are then quantified by employing high-speed videography combined with a three-dimensional micro-force measurement system. Subsequently, a theoretical model is developed to investigate the relationships between the jumping force and performance of *V. micado*, focusing on the effects of the jumping force, gravity, aerodynamic drag, and take-off angle on the jumping performance. Our model suggests there exist jumping strategies that facilitate take-off with greater power output while minimizing take-off displacement and acceleration time. This study lays a foundation to understand the jumping mechanisms of insects and may provide inspiration for the design of high-performance jumping robots and catapult systems.

## 2. Materials and Methods

### 2.1. Experiments

**Figure 1:**
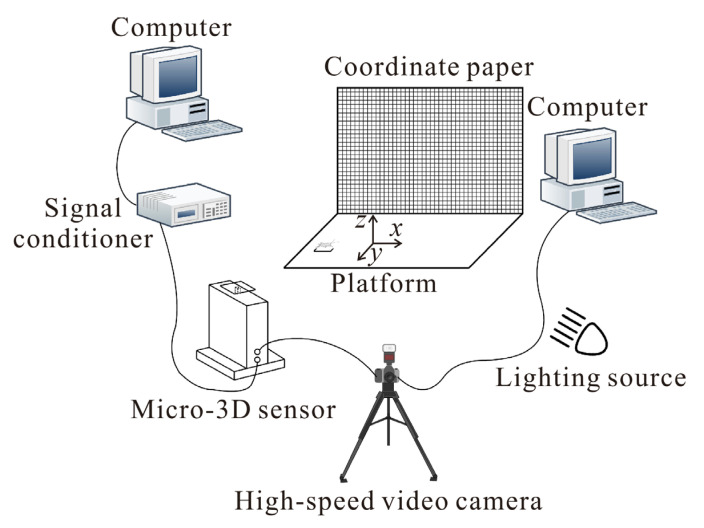
Schematic diagram of the experimental setup for motion observation and force measurement [17].

Crickets *Velarifictorus micado* (Orthoptera, Gryllidae) were collected from fields in Shandong Province, China. The experimental system for recording cricket jumping consisted of a motion observation setup and a 3D micro-force measurement system (Figure 1) [17]. Images of jumping movements were captured at 1000 frames per second by using a high-speed video camera (FASTCAM 1024PCI, Photron Ltd., Tokyo, Japan) on the right side of the insects. These images were stored directly on a computer and analyzed with MATLAB R2021b (The MathWorks, Inc., Natick, MA, USA) and Microsoft Visio 2019 (Microsoft Corporation, Redmond, WA, USA) to measure the kinematics. The 3D jumping force, including fore–aft force (forward or backward component of the jumping force) *F*_x_, lateral force *F*_y_, and normal force *F*_z_, were measured by using a force measurement sensor [33,34]. The load limit of the 3D sensor is 1500 mN and the load resolution in the x-, y- and z-directions are 2, 2, and 3 mN, respectively [33]. A thin glass load carrier (20 mm × 20 mm × 0.8 mm) coated with P360 sandpaper (average particle size ≈ 40.5 μm) was glued to the 3D sensor, providing a flat and stable substrate for crickets. The selected sandpaper offered a moderate surface roughness that allowed the insects to obtain a firm grip [35], and no slipping was observed during the jumping experiments. The natural frequencies of the system (sensor with load carrier) were above 125 Hz, which was high enough to avoid any influence on the measured results. The jumps of crickets were spontaneous or encouraged by touching the cercus lightly with a fine paintbrush. According to the 3D jumping force, the azimuth angle of jumping can be determined by the momentum theorem. Only jumps that were parallel or nearly parallel to the image plane of the camera were considered, based on the criterion that the azimuth angle was less than 10°.

Jumping tests had been performed at temperatures of 28–32 °C and humidity of 50–70%. A minimum of three movements was recorded for each insect, and movements were evoked at approximately 5 min intervals to allow the crickets to recover before the next attempt. 56 jumps performed by 14 adult males and 20 jumps by 5 females *V. micado* were selected for analysis. The morphology of the hind legs was photographed by using a stereomicroscope fitted with a Microvision MV-130UC digital camera.

To compare different jumps, the initial take-off time (*t* = 0 ms) was defined as the moment when the jumping force was generated. The acceleration time was defined as the interval from the initial extension of the fully flexed hind legs to take-off. The *g*-force, defined as the ratio of jumping acceleration to gravitational acceleration, is used to evaluate the jumping performance of insects. The take-off (launch) velocity was measured in the final millisecond before losing contact with the substrate. The take-off angle was defined as the angle between the horizontal and the line connecting the center-of-mass (COM) positions at the first four frames after take-off. Jump displacements were measured by the motion of the COM that was determined by using MATLAB code. SPSS 26.0 (IBM Corporation, Armonk, NY, USA) was used for statistical analysis on all experiments. All data were determined to be normally distributed by using the Shapiro–Wilk test. Data are presented as the mean of individual means ± standard error of the mean (SEM), which is commonly used in studies on jumping performance of insects [25,36,37]. For each individual cricket, the mean value of all repeated jumps was first calculated to obtain an individual mean. These individual means were then used as independent samples for statistical analysis. This method avoids pseudoreplication caused by repeated measurements within the same individual.

### 2.2. Theoretical Model

**Figure 2:**
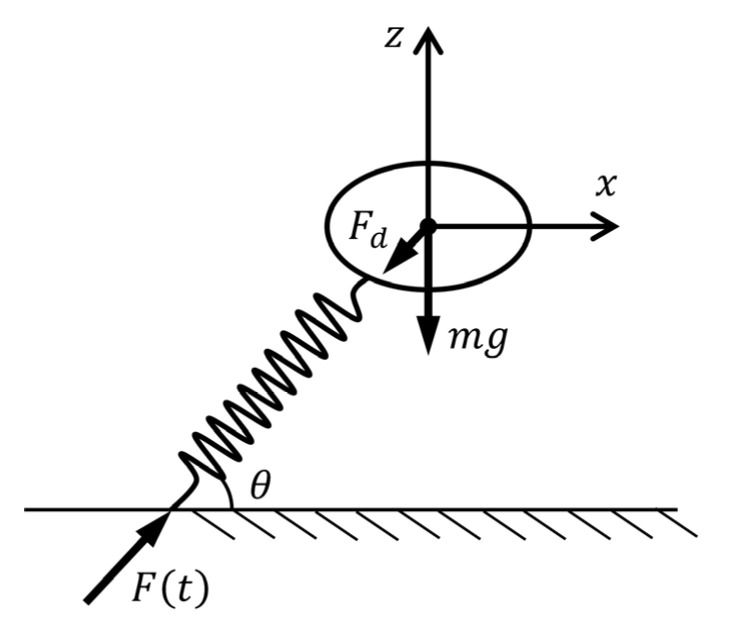
Illustration of the theoretical model.

The jumping process of crickets can be divided into the take-off phase and the aerial phase. A comprehensive theoretical framework for the aerial phase has been established in our previous work [17]. In this section, we developed a theoretical model to describe the jumping performance of crickets during the take-off phase, incorporating the effects of the jumping force, gravity, aerodynamic drag, and take-off angle (Figure 2). The take-off motion is approximated using a mass–spring system, where a massless spring simulates the time-dependent jumping force *F*(*t*). The governing equations are

(1) $$\left\{\begin{array}{l} m\ddot{x}\left(t\right)=F\left(t\right)\cos\theta -k{\dot{x}}^{2}\left(t\right)\\ m\ddot{z}\left(t\right)=F\left(t\right)\sin\theta -mg-k{\dot{z}}^{2}\left(t\right) \end{array}\right.,$$

where m is the body mass, g is the gravitational acceleration, θ is the take-off angle, and $k=\rho A_{d}C_{d}/2$ is the aerodynamic drag constant, which is a function of air density ρ, frontal area $A_{d}$, and drag coefficient $C_{d}$. In Equation (1), the aerodynamic drag is proportional to the square of the velocity. This relation arises from the physical balance between inertial forces and viscous forces in a moving fluid, which is characterized by the Reynolds number. When the Reynolds number is lower, drag is due mainly to viscosity and is proportional to the velocity. For larger Reynolds numbers, drag is due mainly to the kinetic energy given to the fluid and is proportional to the square of velocity. The crickets move through the air with Reynolds numbers high enough for inertial forces to dominant [17]. Therefore, the assumption that aerodynamic drag is proportional to the square of the velocity.

The instantaneous jumping force produced by the hind legs was simulated by a skewed generalized Gaussian function:

(2) $$F\left(t\right)=Ae^{\left[-{\left|\frac{t-\mu }{\tau \left(1+q\times \mathrm{sgn}\left(t-\mu \right)\right)}\right|}^{p}\right]},$$

where A is the peak magnitude, μ is the peak time, τ is the characteristic duration, p is the shape factor, which controls the pulse sharpness, and q is the skewness factor, which controls the asymmetry. When q>0, the jumping force produces a fast rise and slow decay (front sharp-loaded) profile, whereas q<0 yields a gradual rise and sharp drop (front slow-loaded) profile. The skewed generalized Gaussian function allows independent control of shape and asymmetry through its parameters, enabling precise fitting of experimental force–time data. This capability allows the model to accurately reproduce the real shape of the jumping force and, importantly, to reveal how the degree of asymmetry in the force profile influences jumping performance. To systematically analyze the contributions of the jumping force, gravity, aerodynamic drag, and take-off angle, we reformulate the cricket take-off equations in nondimensional form. Firstly, we introduce nondimensional variables:

(3) $$\xi =\frac{x}{L_{0}},$$

(4) $$\zeta =\frac{z}{L_{0}},$$

where $L_{0}$ is the characteristic length, which is determined by a typical jumping force of magnitude $A_{0}$ over time $t_{0}$:

(5) $$L_{0}=\frac{A_{0}t_{0}^{2}}{m}.$$

The velocity and acceleration can be expressed as

(6) $$\left\{\begin{array}{l} \dot{x}=\frac{L_{0}}{t_{0}}\frac{d\xi }{d\tau }\\ \dot{z}=\frac{L_{0}}{t_{0}}\frac{d\zeta }{d\tau } \end{array}\right.,$$

(7) $$\left\{\begin{array}{l} \ddot{x}=\frac{L_{0}}{t_{0}^{2}}\frac{d^{2}\xi }{d\tau^{2}}\\ \ddot{z}=\frac{L_{0}}{t_{0}^{2}}\frac{d^{2}\zeta }{d\tau^{2}} \end{array}\right.,$$

respectively.

Substituting Equations (3)–(7) into Equation (1) yields

(8) $$\left\{\begin{array}{l} m\frac{L_{0}}{t_{0}^{2}}\frac{d^{2}\xi }{d\tau^{2}}=F\left(t\right)\cos\theta -k\frac{L_{0}^{2}}{t_{0}^{2}}{\left(\frac{d\xi }{d\tau }\right)}^{2}\\ m\frac{L_{0}}{t_{0}^{2}}\frac{d^{2}\zeta }{d\tau^{2}}=F\left(t\right)\sin\theta -mg-k\frac{L_{0}^{2}}{t_{0}^{2}}{\left(\frac{d\zeta }{d\tau }\right)}^{2} \end{array}\right..$$

Dividing Equation (8) by ${mL}_{0}/t_{0}^{2}$ normalizes the inertial terms, and Equation (8) becomes

(9) $$\left\{\begin{array}{l} \frac{d^{2}\xi }{d\tau^{2}}=\frac{F\left(t\right)t_{0}^{2}}{mL_{0}}\cos\theta -\frac{kL_{0}}{m}{\left(\frac{d\xi }{d\tau }\right)}^{2}\\ \frac{d^{2}\zeta }{d\tau^{2}}=\frac{F\left(t\right)t_{0}^{2}}{mL_{0}}\sin\theta -\frac{gt_{0}^{2}}{L_{0}}-\frac{kL_{0}}{m}{\left(\frac{d\zeta }{d\tau }\right)}^{2} \end{array}\right..$$

By substituting Equation (5) into Equation (9), the nondimensional equation of take-off becomes

(10) $$\left\{\begin{array}{l} \frac{d^{2}\xi }{d\tau^{2}}=f\left(\tau \right)\cos\theta -\gamma {\bar{v}}_{\xi }^{2}\\ \frac{d^{2}\zeta }{d\tau^{2}}=f\left(\tau \right)\sin\theta -\lambda -\gamma {\bar{v}}_{\zeta }^{2} \end{array}\right..$$

The normalized drag factor and gravity factor are defined as

(11) $$\gamma =\frac{kA_{0}t_{0}^{2}}{m^{2}},$$

(12) $$\lambda =\frac{mg}{A_{0}},$$

respectively. The normalized jumping force is expressed as

(13) $$f\left(\tau \right)=\bar{A}e^{\left[-{\left|\frac{\bar{t}-\bar{\mu }}{\bar{\tau }\left(1+q\cdot \mathrm{sgn}\left(\bar{t}-\bar{\mu }\right)\right)}\right|}^{p}\right]},$$

where

(14) $$\bar{A}=\frac{A}{A_{0}},$$

(15) $$\bar{t}=\frac{t}{t_{0}},$$

(16) $$\bar{\mu }=\frac{\mu }{t_{0}},$$

(17) $$\bar{\tau }=\frac{\tau }{t_{0}}.$$

the normalized resultant velocities $\bar{v}$ and displacements $\bar{s}$ can be calculated as

(18) $$\bar{v}=\sqrt{v_{\xi }^{2}+v_{\zeta }^{2}},$$

(19) $$\bar{s}=\sqrt{\xi^{2}+\zeta^{2}},$$

respectively.

From Equations (10)–(19), the take-off velocities ($v_{\xi }$, $v_{\zeta }$, and $\bar{v}$) and displacements (ξ, ζ, and $\bar{s}$) in the fore–aft, normal, and resultant directions, respectively, can be determined.

The jumping power output can be calculated as

(20) $$\bar{P}=f\left(\tau \right)\times \bar{v}.$$

## 3. Results

### 3.1. Body Morphology

**Figure 3:**
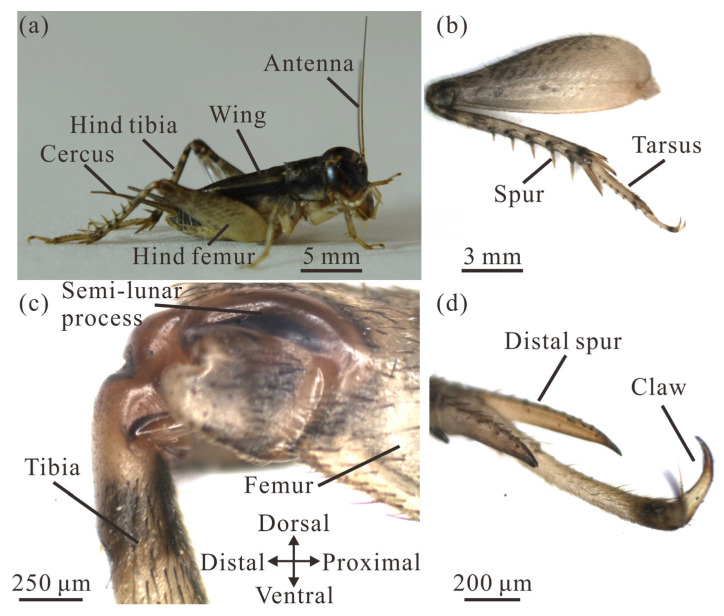
Morphology of the cricket *V. micado*. (**a**) The body structure. (**b**) The hind leg. (**c**) The hind femur–tibia joint. (**d**) Distal structures of the tarsus.

Adult *V. micado* exhibits an elongated body with a pair of long and robust hind legs (Figure 3a). Males have an average body mass of 368 ± 11 mg, which is significantly 41% lighter than the mass of adult females (624 ± 35 mg) (*t* = −9.56, *p* < 0.001). The body length of 17.05 ± 0.18 mm for adult males (excluding the cerci) is significantly 13.9% shorter than that of adult females (19.81 ± 0.21 mm) (*t* = −8.22, *p* < 0.001). The membranous hind wings, responsible for producing acoustic signals, remain inactive and do not flap during jumping [17].

The hind legs of male and female *V. micado* are 155% and 153% the length of their bodies. The length of the hind leg of males is 26.44 ± 0.13 mm (including tarsi), which is significantly 12.9% shorter than that of 30.37 ± 0.09 mm for female *V. micado* (*t* = −23.14, *p* < 0.001). When normalized to the length of the tarsus, the hind leg segmental proportions are 1: 1.35: 1.83 (tarsus: tibia: femur) for males and 1: 1.33: 1.77 for females, indicating a progressively elongating structure from distal to proximal segments (Figure 3b). The hind femur of the crickets is more than twice the length of the middle and fore femora, and its maximum width is approximately three times greater than that of the femora on the other legs. The surfaces of the femur and tibia are described as dorsal, ventral, distal and proximal with reference to their position, as shown in Figure 3c. Each distal hind femur has one pair of semi-lunar processes that are composed of heavily sclerotized, hard cuticle [38,39,40]. The hind tibia of male *V. micado* is 8.53 ± 0.08 mm long and is 13.3% shorter than that of females, which is 9.84 ± 0.04 mm long, representing a significant difference (*t* = −13.86, *p* < 0.001). For males, two dorsal spur rows ranging from 0.73 to 1.95 mm in length are present along the hind tibia (Figure 3b). The hind tarsus is 6.31 ± 0.04 mm long in males and is 14.7% shorter than that of females, which is 7.40 ± 0.04 mm, representing a significant difference (*t* = −18.98, *p* < 0.001). At its distal end, a pair of claws and spurs (Figure 3d), together with the distal tibial spurs, enhance ground purchase and contact stability during jumping.

### 3.2. Take-Off Movement

**Figure 4:**
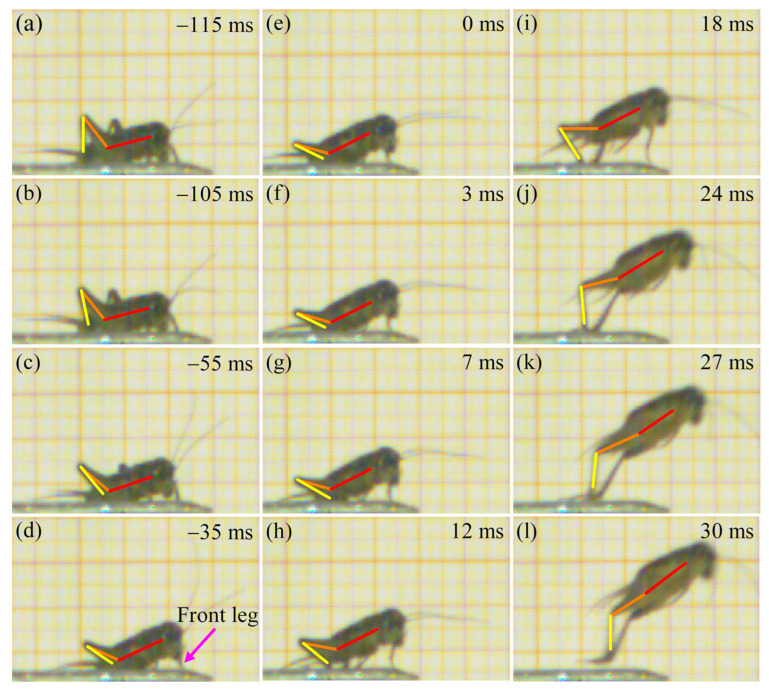
High-speed images of a jump by a male *V. micado*, where the red line represents the body axis, while the orange line and the yellow line represent the femoral axis and the tibial axis, respectively. (**a**) −115 ms. (**b**) −105 ms. (**c**) −55 ms. (**d**) −35 ms. (**e**) 0 ms. (**f**) 3 ms. (**g**) 7 ms. (**h**) 12 ms. (**i**) 18 ms. (**j**) 24 ms. (**k**) 27 ms. (**l**) 30 ms.

**Figure 5:**
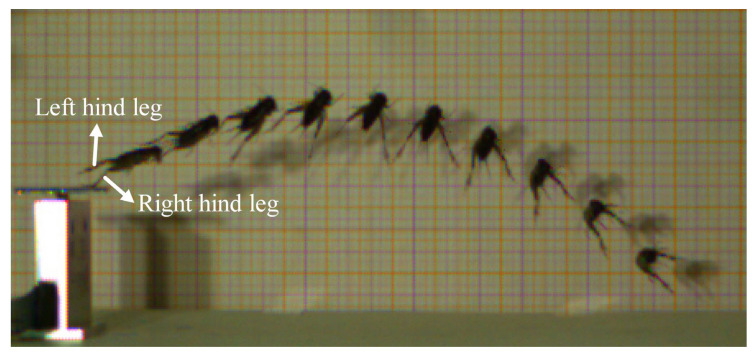
The sequential high-speed image of a typical rotational movement of *V. micado* after take-off.

Prior to take-off, the orientation of the body is adjusted through coordinated leg movements. The hind tibiae gradually flex and rotate around the femora until they are fully appressed (Figure 4a–c). Subsequently, the front legs extend to elevate the anterior portion of the body, increasing the body-ground angle. Concurrently, the hind legs rotate downward at the coxal joints (Figure 4d). Once the desired body posture is achieved (Figure 4e), the tibiae rapidly extend around the femora (Figure 4f–k). This powerful extension of the hind legs propels the body upward until all legs lose contact with the substrate (Figure 4l). The aerial phase begins immediately after the legs lose contact with the substrate, during which the *V. micado* follows a ballistic trajectory determined by the take-off conditions. Specifically, the position, velocity, angle, and body orientation at the final moment of the take-off phase serve as the initial conditions for the aerial phase. Therefore, the mechanical behavior during take-off has a critical influence on the jumping performance in the aerial phase. Our previous study revealed that crickets exhibited distinct rotational movements during this airborne stage, including pitching, yawing, rolling, and their combinations [17]. These rotations primarily arise from asymmetry in the jumping force generated by the hind legs. As shown in Figure 5, the hind legs do not extend synchronously during take-off phase, causing the thrust vector to deviate from the COM and inducing significant rotation. The body rotation, together with the effect of the aerodynamic drag, increase the indeterminacy and unpredictability of jumping trajectories, which is advantageous for escaping from predators [41,42].

**Figure 6:**
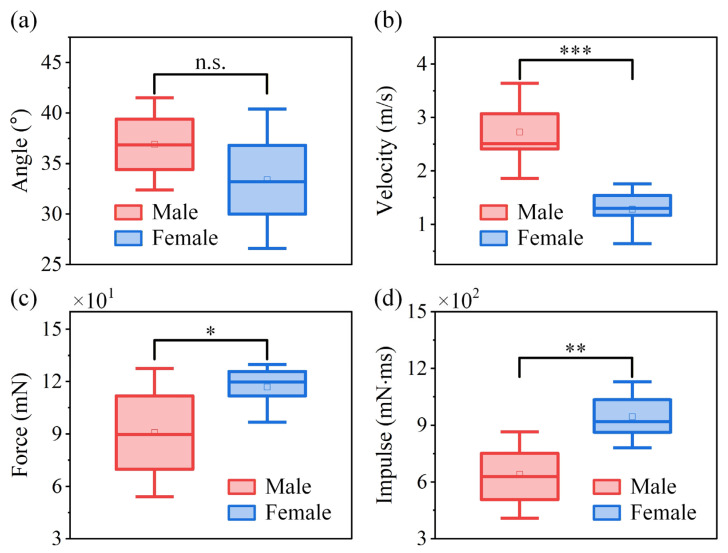
Box plots of individual means comparing (**a**) take-off angle, (**b**) take-off velocity, (**c**) jumping peak force, and (**d**) jumping peak impulse between males and females *V. micado*. Boxes indicate the interquartile range (IQR), the horizontal line inside each box represents the median, the square marker denotes the mean value, whiskers show 1.5 × IQR, and points outside the whiskers are plotted as outliers. Statistical significance between males and females is indicated as n.s. (not significant, *p* > 0.05), * (*p* < 0.05), ** (*p* < 0.01), and *** (*p* < 0.001).

Figure 6 presents the statistical data based on the primary jumping performance, including take-off angle, take-off velocity, jumping peak force, and jumping peak impulse for both sexes. The take-off angle of *V. micado* ranges from 11.8° to 53.1° in jumps by both sexes. The take-off angle of the trajectory is 36.9 ± 0.79° in male *V. micado* and is 33.4 ± 2.4° in females. The independent two-sample *t*-tests demonstrate that males and females show no significant difference in take-off angle (Figure 6a), indicating that both sexes adopt similar jumping strategies. However, males exhibit a significantly higher take-off velocity (Figure 6b) despite having significantly lower jumping peak force (Figure 6c) and impulse (Figure 6d) than females, which suggests that males are able to achieve greater power output during take-off. The take-off performance of adult male and female *V. micado* is summarized in Table 1. The extension time of a jump in males is 27.1 ± 0.39 ms, and in females, it is 28.2 ± 0.86 ms, showing no significant difference between these values. The normal, fore–aft, and resultant jumping peak forces generated by adult males are 60.1 ± 4.76 mN, 67.2 ± 4.81 mN, and 90.8 ± 6.51 mN, respectively. In adult females, the values are 74.4 ± 5.68 mN, 88.7 ± 5.80 mN, and 116.8 ± 5.85 mN, respectively. In the best jump, the resultant jumping peak force is 170.3 mN in males with experienced forces of 56 *g*, and the resultant jumping peak force is 230 mN in females with experienced forces of 30 *g*.

We also conduct statistical analysis of all individual jumping data (Figure S1, Supplementary Materials). Although a few outliers are present, all datasets satisfy the Shapiro–Wilk normality test. Importantly, consistent with the statistical analysis based on individual means, independent two-sample *t*-tests on the full data of all individual jumps also reveal no significant sex difference in take-off angle (Figure S1a), significantly higher take-off velocity in males (Figure S1b), and significantly lower peak force (Figure S1c) and impulse (Figure S1d) compared with females.

The energy required to achieve these take-off performances can be calculated as

(21) $$E=mgh+\frac{1}{2}mv^{2}.$$

where *h* is the measured height variation in the crickets’ center of mass (COM) in the final millisecond before they lose contact with the substrate, and *v* is the take-off velocity.

The jumps by males require an energy of 1297 μJ, and those by females require 1699 μJ. We measured that the mass of the hind legs of *V. micado* account for about 11% of body mass, which is same as the previous assumption [21]. The jumps by males require a power output per mass of muscle of 1874 W/kg, while in females, the power output per mass of muscle was lower at 1360 W/kg. The results indicate that *V. micado* might employ a catapult-mechanism, because the power output per mass of muscle exceed the maximum active contractile limit of 250 to 500 W/kg for normal muscle [43,44,45,46]. However, the energy storage and release mechanisms remain unclear.

### 3.3. Verification of the Theoretical Model

**Figure 7:**
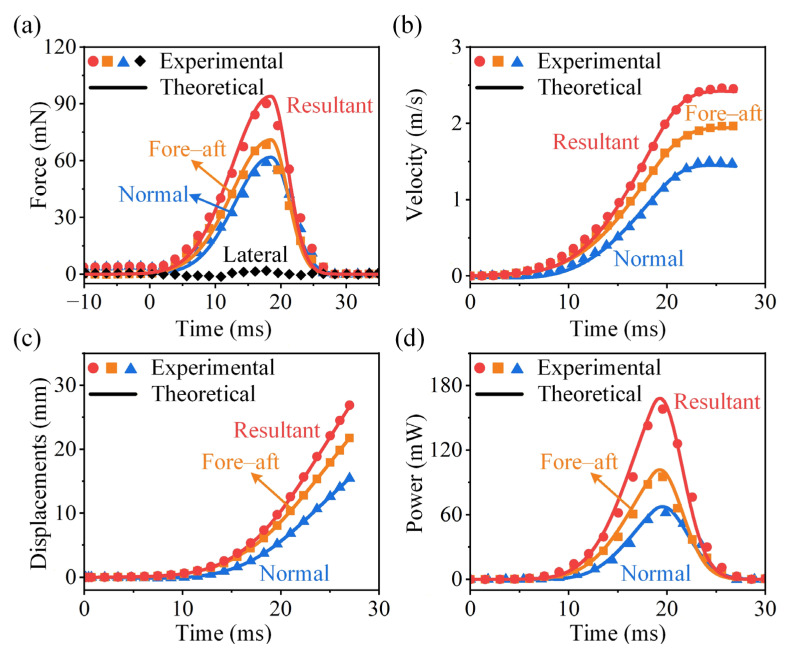
Experimental measurements and theoretical predictions of the take-off performance of the cricket *V. micado*. Jumping (**a**) force, (**b**) velocity, (**c**) displacement, and (**d**) power as functions of time.

In this section, the theoretical model is validated with the experiments. We compare the theoretical and experimental results for the jumping force, velocity, displacement, and power output during the cricket *V. micado* take-off, including fore–aft, normal, and resultant components. In this example, A = 94 mN, μ = 18.4 ms, p = 2, and q = −0.4. The drag coefficient $C_{d}\;=\;0.3$ is adopted [17]. As a median example, Figure 7 shows that the theoretical predictions are in good agreement with the experimental measurements. As males *V. micado* exhibit superior jumping performance, we have additionally included the maximum (Figure S2) and minimum (Figure S3) jump cases in the Supplementary Materials. The comparison across these three representative conditions (maximum, median, and minimum) further demonstrates the correctness of the theoretical model. Notably, we observe that greater vertical (z-direction) jumping forces lead to earlier take-off from the substrate.

As shown in Figure 7a, the skewed generalized Gaussian function accurately predicts both the shape and peak value of the jumping force. The profile exhibits a gradual rise followed by a sharp drop (front slow-loaded profiles), with the peak force occurring approximately two-thirds into the take-off phase. This trend is consistent with observations in locust jumps [47]. It should be noted that the lateral force *F*_y_ was relatively small and had a negligible effect on the resultant jumping force. Therefore, only the fore–aft and normal components are mainly analyzed.

The velocity profiles reveal a non-uniform acceleration pattern, as evidenced by the S-shaped velocity–time curve (Figure 7b). It indicates a progressive increase in acceleration during the tibiae extension, followed by a gradual reduction in acceleration as the hind legs near full extension. The maximum velocity is reached almost at the moment of take-off. The fore–aft velocity remains nearly constant toward the end of take-off, indicating that aerodynamic drag has little effect. In contrast, the vertical velocity slightly declines due to the absence of the propulsive force and the increasing influence of gravity. After take-off, *V. micado* enters the aerial phase, during which the motion is primarily influenced by gravity and aerodynamic drag [17]. The trend of take-off displacements reflects the cumulative effect of velocities (Figure 7c). The power output follows a similar pattern to the jumping force. The maximum power, approximately 180 mW, occurs near the moment of peak force generation (Figure 7d).

## 4. Discussion

### 4.1. Effect of Force Magnitude

**Figure 8:**
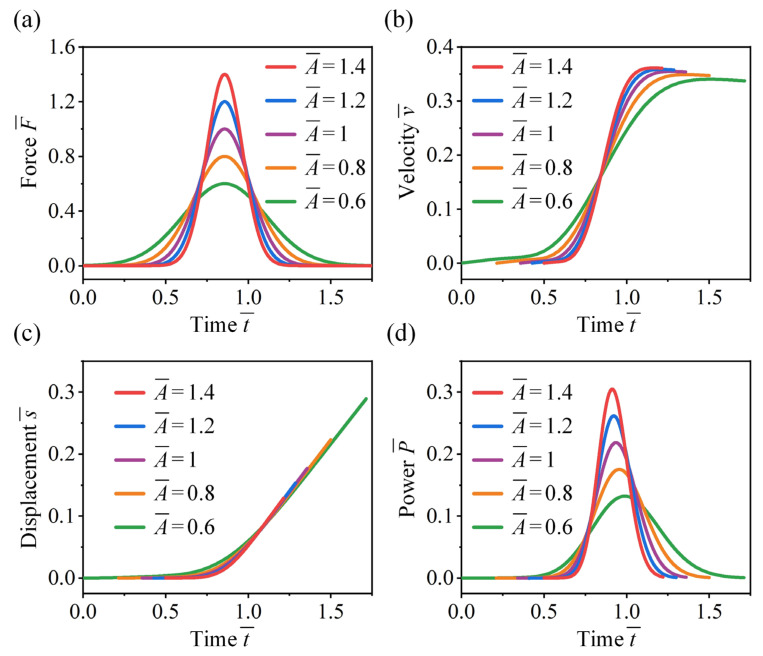
Effect of force magnitude on the take-off performance of the cricket jumping, where $\;\bar{\mu \;}=\;0.8$, p = 2, q = 0, and different $\bar{A}$ values are considered. Normalized jumping (**a**) force, (**b**) velocity, (**c**) displacement, and (**d**) power as functions of $\bar{t}$.

We first analyze the effect of jumping force magnitude on the take-off performance of crickets. As shown in Figure 8a, symmetric jumping forces centered at $\bar{\mu }\;=\;0.8\;$ are considered, with all cases having equal jumping impulse (defined as the area under the force–time curve). As the magnitude increases, the force duration decreases accordingly. Figure 8b shows that higher force magnitudes result in a more rapid increase in take-off velocity, while lower magnitudes with longer durations lead to slower acceleration. The peak velocity exhibits a slight decrease when the force magnitude reduces from 1.4 to 0.6, which is primarily attributed to the increased influence of gravity over prolonged take-off durations. As shown in Figure 8c, the displacement–time trajectories exhibit little variation with changes in the magnitude and duration of the jumping force. However, higher force magnitudes enable the cricket to complete take-off within a shorter displacement range. As shown in Figure 8d, the temporal profile of power output closely resembles that of the jumping force. An increase in force magnitude elevates the peak power and advances its occurrence within the take-off phase.

### 4.2. Effect of Force Skewness

**Figure 9:**
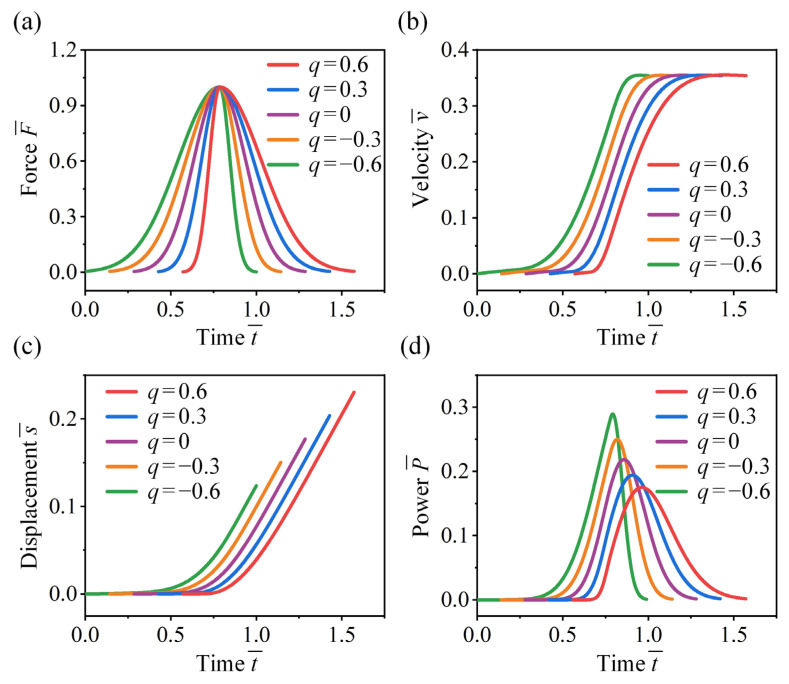
Effect of force skewness on the take-off performance of the cricket jumping, where $\bar{\mu \;}=\;0.75$, p = 2, $\bar{A}\;=\;1$, and different q are considered. Normalized jumping (**a**) force, (**b**) velocity, (**c**) displacement, and (**d**) power as functions of $\bar{t}$.

Now we examine the effect of force skewness on the take-off performance of crickets. In Figure 9a, jumping forces with equal impulse and characteristic duration are considered, where peak magnitude $\bar{A}\;=\;1$, peak time $\bar{\mu }\;=\;0.75$, and varying skewness factors q are adopted. Figure 9b shows that while skewness has little influence on the final take-off velocity, it significantly affects the growth rate of velocity. A front sharp-loaded force (q > 0) leads to greater acceleration, while a front slow-loaded force (q < 0) results in a more gradual increase in velocity. It is observed that the front slow-loaded force yields a lower average velocity, enabling the insect to complete take-off over a shorter displacement (Figure 9c). Specifically, the take-off displacement for q = −0.6 is 40% lower than that for q = 0.6.

Furthermore, force skewness has a significant effect on the instantaneous power output (Figure 9d). Although front sharp-loaded forces generate higher acceleration, they result in lower power output. A higher instantaneous power output can be achieved when both the jumping force and the corresponding velocity reach larger values simultaneously. For instance, when the skewness factor is q = −0.6, the power output is 67% higher compared to q = 0.6, and the peak time is advanced by 17.6%. These results explain why both crickets and locusts adopt a front slow-loaded force during take-off, which facilitates take-off with greater power output while minimizing take-off displacement and acceleration time.

### 4.3. Effect of Gravity Factor

**Figure 10:**
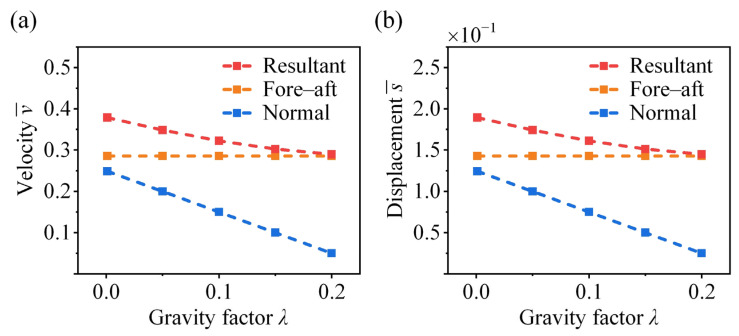
Effect of gravity factor on the take-off (**a**) velocity and (**b**) displacement of the cricket jumping, where $\bar{\mu }\;=\;0.5$, p = 2, q = 0, $\bar{A}\;=\;1$, γ = 0, θ = 42°, and different λ are considered.

We conduct a parametric analysis to evaluate the effect of gravitational loading on the jumping performance of crickets, where different gravity factors are considered. It can be found that both the normal and resultant take-off velocities decrease with an increasing gravity factor λ, while the horizontal velocity remains unaffected (Figure 10a). A similar decreasing trend is observed for the vertical and resultant displacements in Figure 10b, as gravitational loading primarily influences the vertical propulsive component. In the face of varying gravitational environments, the optimal take-off performance of bio-inspired jumping robots may require distinct force profiles adapted to the specific gravitational level.

### 4.4. Effect of Drag Factor

**Figure 11:**
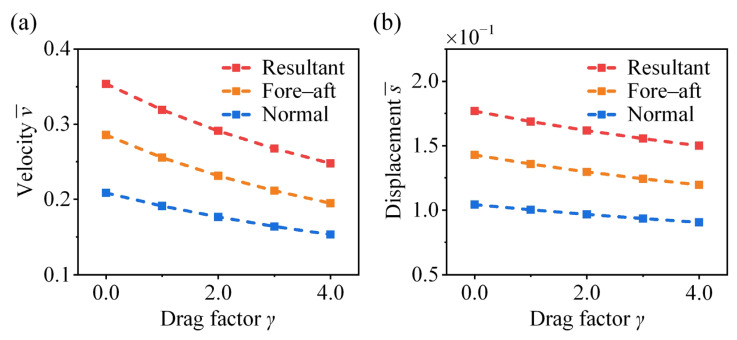
Effect of drag factor on the take-off (**a**) velocity and (**b**) displacement of the cricket jumping, where $\bar{\mu }\;=\;0.5$, p = 2, q = 0, $\bar{A}\;=\;1$, λ = 0.041, θ = 42°, and different γ values are considered.

As shown in Figure 11, the velocities and displacements in fore–aft, normal, and resultant directions decrease monotonically with increasing the drag factor γ. In our previous work, the effect of aerodynamic drag on the post-take-off jumping performance of crickets was investigated, revealing the critical role of the hind legs in posture regulation and drag reduction during aerial motion [17]. Although actual aerodynamic drag exerts a negligible influence during the take-off phase (Figure 7b), reducing its effects can help preserve the original jumping performance. Integration of the present findings with previous results yields a comprehensive understanding of the efficient jumping strategy employed by crickets.

### 4.5. Effect of Take-Off Angle

**Figure 12:**
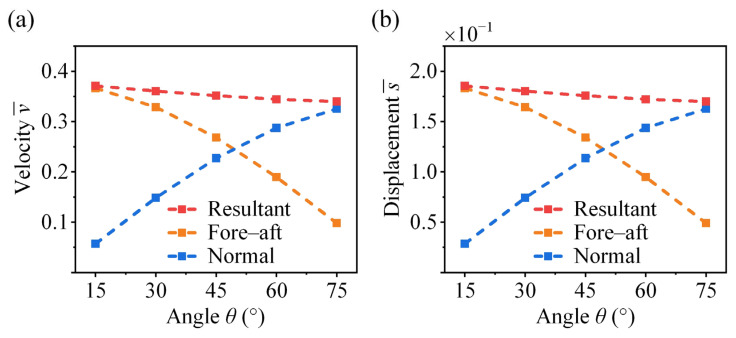
Effect of take-off angle on the take-off (**a**) velocity and (**b**) displacement of the cricket jumping, where $\bar{\mu }\;=\;0.5$, p = 2, q = 0, $\bar{A}\;=\;1$, λ = 0.041, γ = 0, and different θ are considered.

As shown in Figure 12, the fore–aft velocity and displacement exhibit a nonlinear decrease as the take-off angle θ increases, with the rate of reduction becoming larger. In contrast, the normal velocity and displacement increase with θ. However, due to gravitational effects, the rate of increase diminishes at higher angles. The coupled effects of the fore–aft and normal components lead to a slight decrease in the resultant velocity and displacement as θ increases. The take-off angle in insects is associated with their habitat conditions and functional demands. For example, the fighting cricket *V. micado* does not use their wings after take-off, whereas locusts actively deploy their wings to flight. Experimental observations show that locusts exhibit higher take-off angles (above 45°), which are advantageous for initiating wing opening and transitioning to flight [21,47]. In contrast, crickets exhibit lower take-off angles (below 45°), which help reduce aerodynamic drag and improve efficiency during the aerial phase [17].

## 5. Conclusions

In this study, we have investigated the jumping kinematics and performance of the fighting cricket *V. micado* through a combination of experiments and theoretical modeling. The external morphology of the body and hind legs is characterized. Using high-speed imaging and three-dimensional force measurements, the take-off process and jumping performance are analyzed. Subsequently, we propose a theoretical model to decipher the relationships between the jumping force and performance. As demonstrated by experiments, this model can precisely predict the jumping force and behaviors of crickets. The effects of the jumping force, gravity, aerodynamic drag, and take-off angle on the jumping velocity, displacement, and power output of crickets are examined. It is found that a higher force magnitude and a front slow-loaded force both enable the insects to complete take-off within a shorter displacement and time while simultaneously increasing peak power and advancing its occurrence. The mechanistic advantages of jumping force with different profiles are elucidated, and the efficient jumping strategy in insects has been identified. The results show that, in the actual jumping process of crickets, the effect of aerodynamic drag on the take-off velocity is negligible. However, when the drag factor is increased, both the take-off velocity and displacement decrease accordingly. Gravitational loading primarily affects the vertical propulsive component. With the influence of gravity, the coupled effects of the fore–aft and normal components lead to a decrease in the resultant displacement as take-off angle increases. This study not only enhances our understanding of the jumping mechanisms of crickets but also provides valuable guidance for designing high-performance jumping robots.

In the present paper, we have only considered the jumping kinematics and performance in *V. micado*. In fact, crickets have more complex microstructures in their hind legs, and the mechanisms underlying energy storage, release, and the overall jumping process require further investigation.

## Figures and Tables

**Table 1:** Take-off performance of the cricket *V. micado*.

Sexes	Time (ms)	Velocity (m/s)	Angle (°)	Force (mN)	Impulse (mN∙ms)	Best *g*-Force
Male	27.1 ± 0.39	2.61 ± 0.18	36.9 ± 0.79	90.8 ± 6.51	640.6 ± 41.2	56
Female	28.2 ± 0.86	1.28 ± 0.19	33.4 ± 2.4	116.8 ± 5.85	945.7 ± 63.2	30

## Data Availability

The data presented in this study are available upon request from the corresponding author due to privacy.

## Supplementary Materials

The following supporting information can be downloaded at https://www.mdpi.com/article/10.3390/biomimetics11010049/s1, Figure S1: Box plots of all individual jumps comparing (a) take-off angle, (b) take-off velocity, (c) jumping peak force, and (d) jumping peak impulse between males and females V. micado. Boxes indicate the interquartile range (IQR), the horizontal line inside each box represents the median, the square marker denotes the mean value, whiskers show 1.5 × IQR, and points outside the whiskers are plotted as outliers. Statistical significance between males and females is indicated as n.s. (not significant, *p* > 0.05), * (*p* < 0.05), ** (*p* < 0.01), and *** (*p* < 0.001); Figure S2: Experimental measurements and theoretical predictions of the maximum take-off performance of the male V. micado. Jumping (a) force, (b) velocity, (c) displacement, and (d) power as functions of time. (*A* = 172.3 mN, *μ* = 12.9 ms, *p* = 2, and *q* = −0.4); Figure S3: Experimental measurements and theoretical predictions of the minimum take-off performance of the male V. micado. Jumping (a) force, (b) velocity, (c) displacement, and (d) power as functions of time. (*A* = 50.5 mN, *μ* = 15.6 ms, *p* = 2, and *q* = −0.4).
