# Supplementary material for: Jumping Kinematics and Performance in Fighting Crickets Velarifictorus micado
Source: Biomimetics (Basel). 2026 Jan 7;11(1):49. doi: 10.3390/biomimetics11010049 (PMC12838587; doi:10.3390/biomimetics11010049)
Supplement: Supplementary file 1 [file biomimetics-11-00049-s001.zip › biomimetics-3909493-supplementary.pdf]

biomimetics

MDPI

Article

Supplementary Materials for "Jumping Kinematics and Performance in Fighting Crickets *Velarifictorus micado*"

Yun Xing [1], Yan Zhang [1], Yu Yan [1] and Jialing Yang [2,*]

[1] Institute for Advanced Materials and Technology, University of Science and Technology Beijing, Beijing 100083, China
[2] Institute of Solid Mechanics, School of Aeronautic Science and Engineering, Beihang University, Beijing 100191, China
* Correspondence: jlyangbuaa@aliyun.com

Academic Editor(s): Name

Received: date
Revised: date
Accepted: date
Published: 7 January 2026

Citation: Xing, Y.; Zhang, Y.; Yan, Y.; Yang, J. Jumping Mechanics of Fighting Crickets *Velarifictorus micado*. *Biomimetics* **2025**, *10*, x. https://doi.org/10.3390/xxxxx

Copyright: © 2026 by the authors. Licensee MDPI, Basel, Switzerland. This article is an open access article distributed under the terms and conditions of the Creative Commons Attribution (CC BY) license.

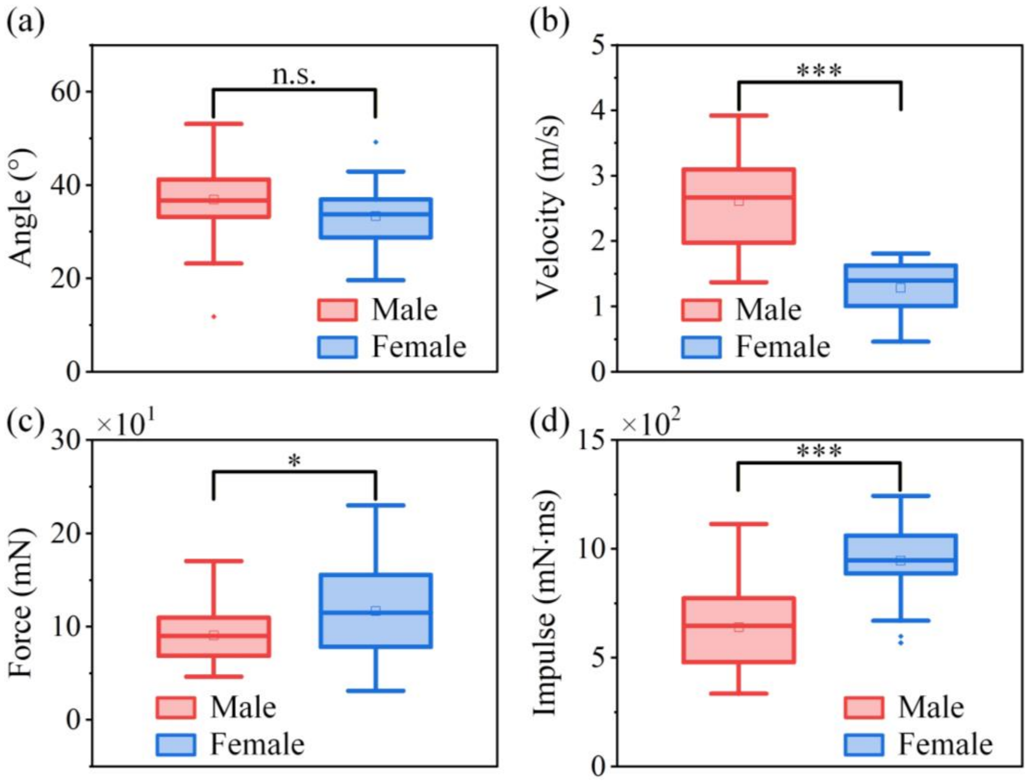

Figure S1. Box plots of all individual jumps comparing (a) take-off angle, (b) take-off velocity, (c) jumping peak force, and (d) jumping peak impulse between males and females *V. micado*. Boxes indicate the interquartile range (IQR), the horizontal line inside each box represents the median, the square marker denotes the mean value, whiskers show 1.5×IQR, and points outside the whiskers are plotted as outliers. Statistical significance between males and females is indicated as n.s. (not significant, P > 0.05), * (P < 0.05), ** (P < 0.01), and *** (P < 0.001).

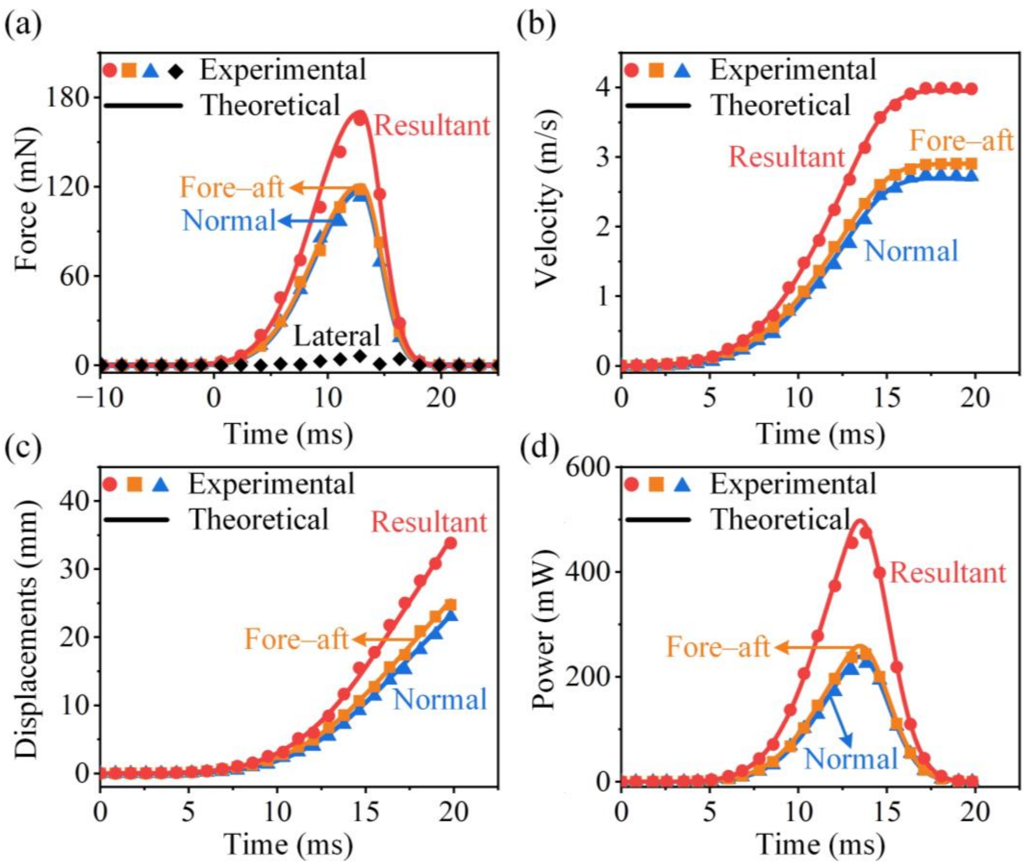

Figure S2. Experimental measurements and theoretical predictions of the maximum take-off performance of the male *V. micado*. Jumping (a) force, (b) velocity, (c) displacement, and (d) power as functions of time. (A=172.3 mN, μ=12.9 ms, p=2, and q=-0.4.)

https://doi.org/10.3390/biomimetics11010049

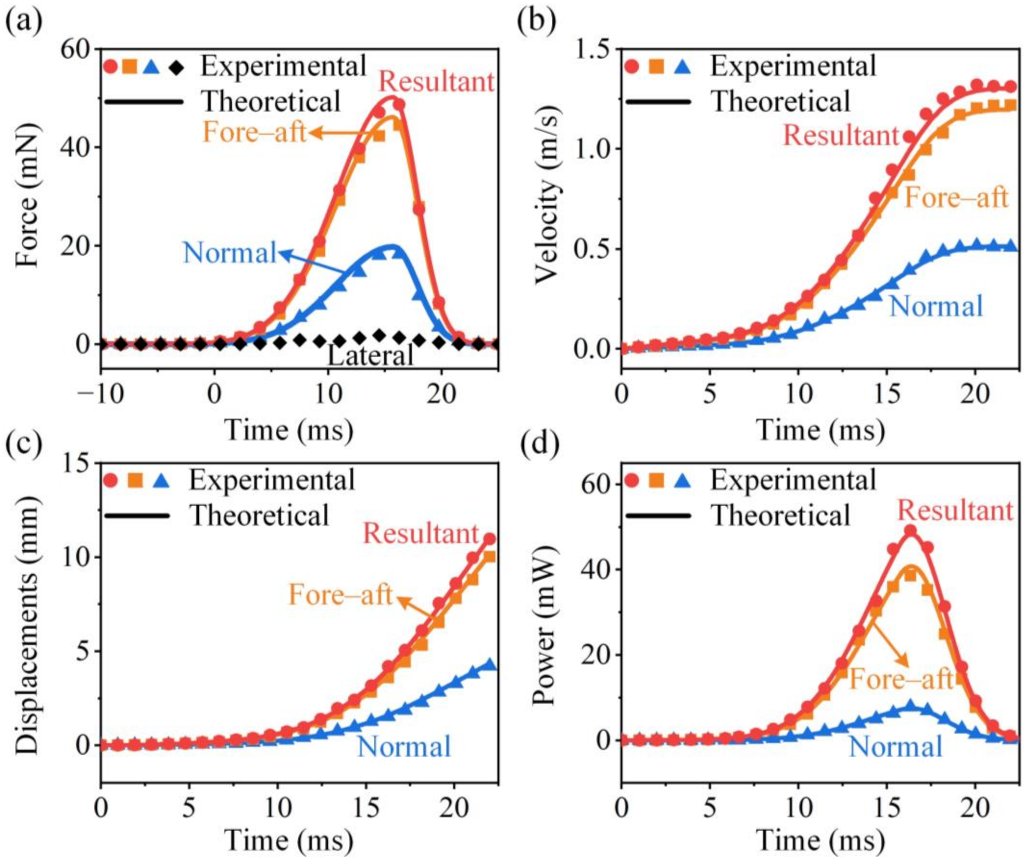

Figure S3. Experimental measurements and theoretical predictions of the minimum take-off performance of the male *V. micado*. Jumping (a) force, (b) velocity, (c) displacement, and (d) power as functions of time. (A=50.5 mN, μ=15.6 ms, p=2, and q=-0.4.)

Disclaimer/Publisher's Note: The statements, opinions and data contained in all publications are solely those of the individual author(s) and contributor(s) and not of MDPI and/or the editor(s). MDPI and/or the editor(s) disclaim responsibility for any injury to people or property resulting from any ideas, methods, instructions or products referred to in the content.

https://doi.org/10.3390/biomimetics11010049
